# An oncolytic HSV-1 vector induces a therapeutic adaptive immune response against glioblastoma

**DOI:** 10.1186/s12967-024-05650-5

**Published:** 2024-09-27

**Authors:** Alberto Reale, Andrea Gatta, Amruth Kaleem Basha Shaik, Mariam Shallak, Anna Maria Chiaravalli, Michele Cerati, Martina Zaccaria, Stefano La Rosa, Arianna Calistri, Roberto Sergio Accolla, Greta Forlani

**Affiliations:** 1 Department of Molecular Medicine, University of Padua, Padua, 35131 Italy; 2 Department of Medicine and Technological Innovation, University of Insubria, via O.Rossi 9, Varese, 21100 Italy; 3 Unit of Pathology, ASST Sette-Laghi, Varese, 21100 Italy

**Keywords:** Glioblastoma, Oncolytic virus, Immunotherapy, HSV-1, GL261

## Abstract

**Background:**

Glioblastoma (GBM) is the most frequent and aggressive brain tumor in adults with the lowest survival rates five years post-diagnosis. Oncolytic viruses (OVs) selectively target and damage cancer cells, and for this reason they are being investigated as new therapeutic tools also against GBM.

**Methods:**

An oncolytic herpes simplex virus type 1 (oHSV-1) with deletions in the γ34.5 neurovirulence gene and the US12 gene, expressing enhanced green fluorescent protein (EGFP-oHSV-1) as reporter gene was generated and tested for its capacity to infect and kill the murine GL261 glioblastoma (GBM) cell line. Syngeneic mice were orthotopically injected with GL261cells. Seven days post-implantation, EGFP-oHSV-1 was administered intratumorally. Twenty-one days after parental tumor challenge in the opposite brain hemisphere, mice were sacrified and their brains were analysed by immunohistochemistry to assess tumor presence and cell infiltrate.

**Results:**

oHSV-1 replicates and induces cell death of GL261 cells in vitro. A single intracranial injection of EGFP-oHSV-1 in established GL261 tumors significantly prolongs survival in all treated mice compared to placebo treatment. Notably, 45% of treated mice became long-term survivors, and rejected GL261 cells upon rechallenge in the contralateral brain hemisphere, indicating an anamnestic antitumoral immune response. Post-mortem analysis revealed a profound modification of the tumor microenvironment with increased infiltration of CD4 + and CD8 + T lymphocytes, intertumoral vascular collapse and activation and redistribution of macrophage, microglia, and astroglia in the tumor area, with the formation of intense fibrotic tissue suggestive of complete rejection in long-term survivor mice.

**Conclusions:**

EGFP-oHSV1 demonstrates potent antitumoral activity in an immunocompetent GBM model as a monotherapy, resulting from direct cell killing combined with the stimulation of a protective adaptive immune response. These results open the way to possible application of our strategy in clinical setting.

**Supplementary Information:**

The online version contains supplementary material available at 10.1186/s12967-024-05650-5.

## Background

Oncolytic viruses (OVs) selectively target cancer cells through various mechanisms, including receptor retargeting [1, 2] or more frequently, exploiting defects in antiviral pathways that are present in cancer cells [3]. Viruses that are either naturally or artificially attenuated in human can often behave as effective OVs. Although many different viruses have been exploited or modified to be used as OVs, to date only one vector, talimogene laherparepvec or T-VEC, based on herpes simplex virus type-1 (oHSV-1) [4], has gained approval for clinical use by the Food and Drug Administration (FDA) and the European Medicines Agency (EMA). T-VEC is engineered with a deletion of both copies of the γ34.5 virulence gene, crucial for causing encephalitis [5]. This deletion blocks both the protein kinase (PKR) pathway activated by dsRNA [6] and autophagy [7]. Additionally, T-VEC features a deletion of the US12 gene, which prevents antigen presentation by infected cells [8]. The deletion of US12 alters the kinetics of another viral gene, US11, allowing it to interfere with the cellular PKR pathway. Thus, the simultaneous deletion of γ34.5 and US12 fine-tunes the virulence of oHSV-1 to enable replication even in moderately resistant cancer cells, while maintaining safety [9]. OVs exert an antitumoral activity not only through direct cytotoxicity of infected cells but also, and importantly, by stimulating an anti-tumor immune response [10]. Notably, the presence of a virus in the tumor microenvironment (TME), typically immunosuppressive, serves as a potent immunogenic stimulus triggering the release of pathogen associated molecular patterns (PAMPs), damage associated molecular patterns (DAMPs) and tumor associated antigens (TAAs) following cancer cell lysis [11].

Glioblastoma (GBM) is a tumor type extensively studied for OV application [12]. GBM, the most common primary brain tumor in adults [13], is characterized by a poor prognosis due to its widespread infiltration and resistance to chemotherapy facilitatedby the intrinsic resistance of drug diffusion due to the blood brain barrier (BBB) [14, 15]. In addition, despite the presence of TAAs, GBM remains largely resistant to current immunotherapeutic approaches including immune checkpoint inhibitors (ICIs) [16, 17] owing to its immunosuppressive TME and poor T cell infiltration [18, 19]. Hence, GBM stands as a promising candidate for oncolytic virotherapy [20], and preclinical and clinical studies are being performed, for example, with several neuroattenuated oHSV1 vectors [21, 22]. Importantly, oHSV-1 can be tested in immunocompetent animal models due to its capacity to infect and replicate in mouse cells as well. Recently, the oHSV-1 G47Δ has been approved for the intratumoral treatment of GBM in Japan [23]. However, to the best of our knowledge, all tested oHSV-1 vectors which retain the virus natural tropism are more attenuated than T-VEC, often featuring a deletion in the ribonucleotide reductase ICP6 gene, that makes the virus incapable of replicating in quiescent cells [24]. This poses a dual challenge, as numerous non-replicating cells are present in the TME [25]. At the same time, GBM cells may exhibit residual antiviral pathway activity, leading to limited activity of highly attenuated vectors. This has been shown in GL261 mouse GBM cells, one of the most common syngeneic immunocompetent models of GBM [26, 27] with a Δγ34.5 oHSV1 [28]. Here we show that GL261 cells are indeed susceptible to infection by an oHSV-1 carrying the same double deletion of γ34.5 and US12 found in T-VEC, and the insertion in the UL55-UL56 intergenic region of the reporter gene enhanced green fluorescent protein (EGFP) (EGFP-oHSV-1) [29]. Following GL261 infection, EGFP-oHSV-1 replicates and induces cytopathic effect and immunogenic cell death (ICD).

To assess the efficacy of EGFP-oHSV-1 treatment in vivo, we investigated the survival rate of mice bearing ann intracranial GL261 tumor when treated with the oncolytic virus. GL261 tumors treated by intracranial injection of EGFP-oHSV-1 exhibited significantly prolonged survival rate accompanied by a drastic modification of the tumor microenvironment when compared to untreated controls. Moreover, long-term survivors displayed resistance to rechallenge with GL261 cells injected into the contralateral brain hemisphere, indicating the presence of an anamnestic antitumoral immune response.

Taken together our findings suggest that a Δγ34.5/Δ US12 oHSV-1 has a stronger antitumoral effect compared to other oHSV-1 backbones in the GL261 syngeneic model [30] and generates an accompanying immune response that ultimately leads to protection from further cancer growth and recurrence. These results are discussed within the frame of novel strategies of immunotherapy against GBM.

## Methods

### Generation of BAC-ΔΔ-EGFP

A bacterial artificial chromosome (BAC) containing the entire genome of HSV-1 strain 17syn+, characterized by a deletion of γ34.5 genes and by the insertion of a cassette expressing the Firefly luciferase (Fluc) under the transcriptional control of the immediate early cytomegalovirus promoter within the intergenic region UL55/UL56 (BAC-Δγ34.5), was kindly provided by Prof. Beate Sodeik (Hannover Medical School) [31]. Starting from BAC-Δγ34.5, the same US12 deletion as described in T-VEC [9] was achieved and the Fluc coding sequence was replaced by a sequence encoding for the enhanced green fluorescent protein (EGFP), as already reported [29]. Briefly, the same US12 deletion as described in T-VEC [9] was achieved, generating BAC-ΔΔ-Fluc. The obtained BAC-ΔΔ-EGFP was used to transfected 293T cells and viral stocks were produced by amplification and titration of the recombinant virus in Vero cells, as previously described [26].

### Cells

Vero cells (green monkey kidney, ATCC CCL-81™), and GL261 cells (mouse glioblastoma, DSMZ ACC802) were maintained in Dulbecco’s modified Eagle medium (DMEM, Gibco, Waltham, MA, USA), supplemented with 1% v/v Penicillin-Streptomycin (Gibco) and 10% v/v fetal bovine serum (FBS) (Gibco). Cells grew in adhesion and were passaged twice a week and cultured at 37 °C, in a 5% CO2 and 98% humidity atmosphere. All cell lines were regularly tested for mycoplasma contamination by endpoint PCR using the AmpliTaq Gold™ DNA polymerase (Applied Biosystems), a forward 50-GGGAGCAAACAGGATTAGATACCCT primer and a reverse 50-TGCACCATCTGTCACTCTGTTAACCTC primer.

### Plaque titration, viability and ATP release assays

Plaque titration assay on Vero cells was performed as described [32]. All infections were carried out by incubating cells for 1 h in a 5% CO2 and 98% humidity atmosphere, in serum-free medium with the appropriate amount of infectious viral particles, based on the desired multiplicity of infection (MOI). Following this incubation, serum-free medium was removed and cells were cultured in medium supplemented with 2% FBS. Cell viability was determined by trypan blue exclusion test. The release of extracellular ATP was measured using RealTime-Glo™ Extracellular ATP Assay (Promega), according to the manufacturer’s instructions. The luminescence signal produced by this assay was analyzed with the Varioskan™ LUX multimode microplate reader.

### Animal models and study design

C57BL/6 (H-2b genotype) female mice aged 6–8 weeks were purchased from Charles River (Charles River Laboratories Italia SRL). The animals were housed for the entire duration of the experiments in the Animal Facility of University of Insubria, in compliance with national regulations on the protection of animals used for scientific purposes (Italian decree n. 26 dated 04/03/2014 acknowledging European Directive 2010/63/EU) and supervised by the animal welfare organism (OPBA). The sample size and power of analysis were determined in terms of formal analysis through the a-priori definition of the underlying effect size using G*power 3.1 software. The sample sizes were obtained by considering, for each experiment, a Type I error rate of 5%, a power of 80%, and an effect size of 0.4. Animals presenting sign of suffering within 7 days after tumor/virus/vehicle injection were excluded from experiment a priori. To minimise potential confounders all the mice were specifically numberd and distributed randomnly in different cages based on the treatment. The total number of animals in each test is reported in the figure legends, and owing to the strategy the number of animals decreased over time. All testings were conducted according to relevant national and international guidelines and were approved by the Italian Ministry of Health (210/2023-PR, 344C6.23; 812/2020-PR; 344C6.16).

### Intracranial tumor injection

All surgical procedures were performed in the animal housing room, following previously established protocols [33]. Prior to surgery, all mice were anesthetized with isoflurane (4% for induction and 1.5% during surgery), and the surgical anesthesia was confirmed by the loss of the pedal reflex. The mice were then positioned in a stereotactic frame, and the skull was cleansed using a sterile cotton swab soaked in 70% ethanol. A 5 mm sagittal incision was made through the scalp, followed by the drilling of a small hole 1.5 mm posterior and 2 mm lateral (right) to the bregma. Subsequently, a total of 3 × 10^4^ GL261 or GL261-CIITA cells in 3 μl of serum-free medium were intracranially injected 3 mm deep from the dura using a 26-gauge needle targeting the right striatum. Seven days post-GL261 tumor injection, two groups of mice were injected in the same brain hemisphere with EGFP-oHSV-1 (from here on abbreviated as oHSV-1) (1 × 10^6^ PFU) or vehicle (PBS). Mice were monitored daily for signs of brain tumor growth, such as seizures, ataxia, or weight loss, and were euthanized if the tumor burden became symptomatic. After 43 days from the injection of the tumor cells, long-term survivor mice were injected in the left striatum with 3 × 10^4^ GL261 parental tumor cells. Subsequently, all mice were euthanized after an additional 3 weeks, and their brains were prepared for histological analysis as described below.

### Immunohistochemistry

The immunohistochemical analysis was conducted in a blinded manner.

All mouse brains were thoroughly sampled, fixed in formalin, and embedded in paraffin. Serial sections of 3-μm thickness were then cut from each paraffin block, mounted on positively charged slides, and stained with hematoxylin and eosin (HE) for morphological evaluation or utilized in immunohistochemical analysis. For immunohistochemistry (IHC), brain sections were deparaffinized, rehydrated, and treated with a 3% hydrogen peroxide solution for 20 min to inhibit endogenous peroxidases. Subsequently, they were washed in TBS with 0.25% Triton X-100 (Sigma Chemical Corp.), and antigen retrieval was performed in a microwave oven at 700-W power for the specified time, using either citrate buffer pH 6 or EDTA buffer pH 8, based on the experimental protocols outlined in Additional File 1.

The tissue sections were then incubated overnight at 4 °C with the specific primary antibody at the working dilution detailed in Additional File 1. The following day, the tissue sections were washed in TBS with 0.25% Triton X-100 and incubated for 45 min at room temperature with the specific biotinylated secondary antibody (Vector). Subsequently, they were incubated for 30 min at room temperature with ABC peroxidase complex (ABC Elite, Vector). The immunoreaction was developed using 3,3′-diaminobenzidine tetrahydrochloride (DAB) (Sigma Aldrich) as a chromogen. After nuclear hematoxylin counterstaining, the tissue sections were dehydrated through an alcohol scale and mounted with a coverslip using Canada balm. The immunostained brain tissues were then analyzed under a light microscope (Olympus). Tumor size and immune infiltration were evaluated as previously described [33].

### Bioinformatic and statistical analysis

Statistical analysis was performed using GraphPad Prism 10 (GraphPad Software, http://www.graphpad.com), and the Student’s t-test was conducted to determine significance. Comparisons were considered statistically significant when the corresponding *p*-value was < 0.05. All data are expressed in the form mean ± standard deviation (SD).

## Results

### oHSV-1 productively infects and induces cell death in murine GL261 cells

**Fig. 1:**
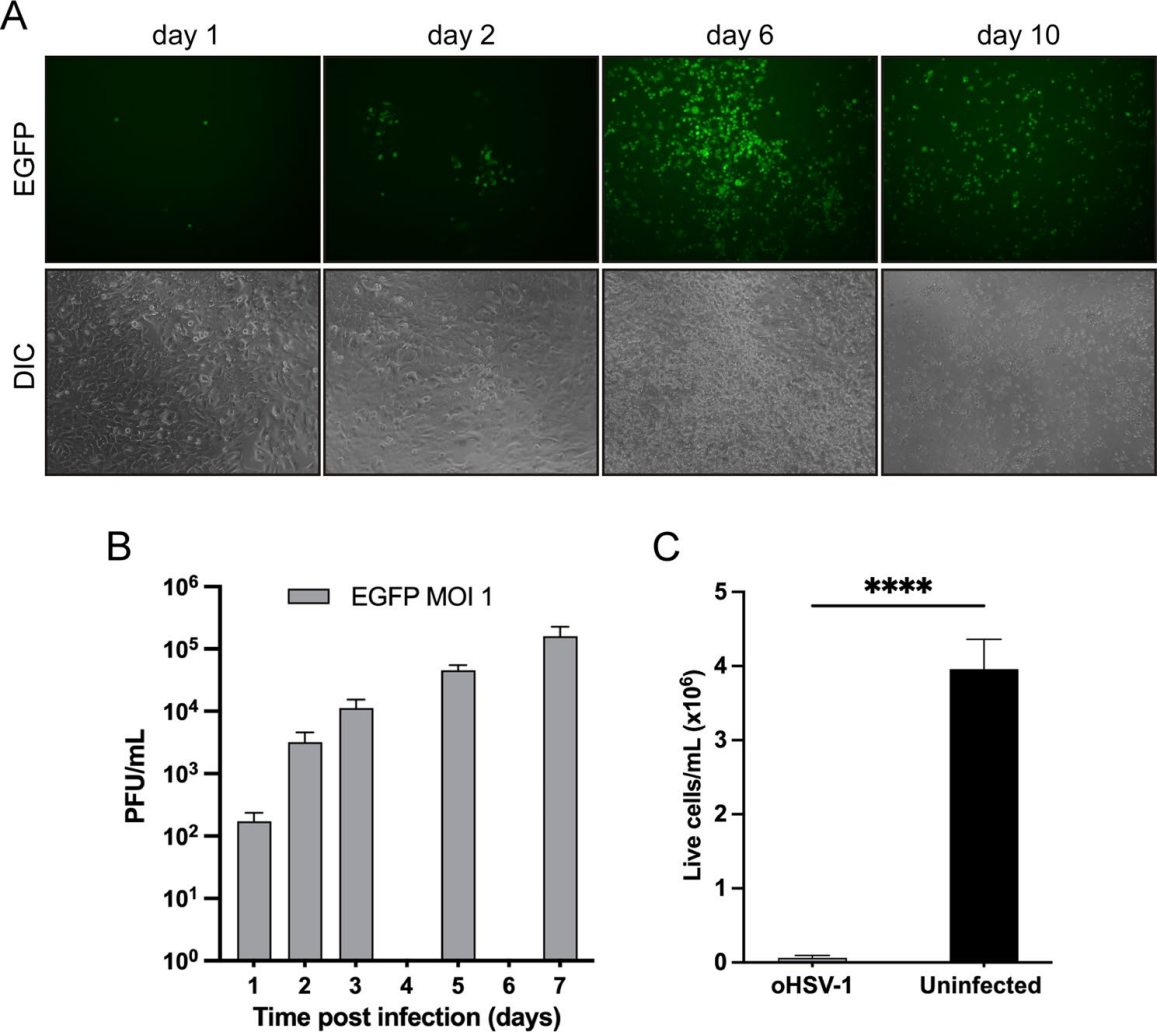
oHSV-1 replicates and induces cell death in GL261 glioblastoma cells in vitro. (**A**) GL261 cells were seeded in a 24-well plate (7.5 × 10^4^ cells/well), infected with oHSV-1 (*MOI = 1*) for 1 h in serum-free DMEM medium, then maintained in DMEM medium supplemented with 2% fetal bovine serum (FBS). The medium was refreshed daily, and EGFP expression, serving as an indicator of viral infection, was monitored at 1, 2, 6, and 10 days post-infection. Fluorescence was monitored using a Leica EpiFluorescence microscope revealing a progressive spread of the infection to the entire monolayer. Concurrently, brightfield microscopy demonstrated an increasing cytopathic effect, characterized by cellular rounding. All images were captured at 10x magnification and represent multiple microscopic fields from a triplicate experiment. (**B**) GL261 cells were seeded in a 24-well plate (7.5 × 10^4^ cells/well), infected with oHSV-1 *(MOI = 1)* for 1 h in serum-free DMEM medium, then maintained in DMEM medium supplemented with 2% FBS. The supernatant was collected and replaced with fresh 2% FBS medium every 24 h. Infectious viral particles were quantified as plaque forming units (*PFU*)/mL by plaque titration assay on green monkey Vero cells. The experiment was performed in triplicate. Y axis is in logarithmic scale. (**C**) 10 days after infection, surviving infected GL261 cells were trypsinized and counted using a hemocytometer and Trypan blue exclusion staining. Uninfected GL261 cells seeded and cultured in the same conditions for 10 days were counted in a similar way. The experiment was performed in triplicate and the difference in the number of live cells between uninfected and oHSV-1-infected GL261 cells was evaluated by Student’s t-test. ****P < 0.0001. In all panels, error bars represent standard deviation

C57BL/6 GL261 mouse cells serve as widely used syngeneic, immunocompetent model of glioblastoma. We infected GL261 cells in vitro with oHSV-1 at a multiplicity of infection (MOI) of 1 plaque forming unit (PFU)/cell. Over time, the fraction of green fluorescent cells gradually increased, along with the onset of cytopathic effects, involving all cells by day 10 post-infection (Fig. 1A). We then quantified viral replication and release of infectious viral particles in the supernatants of GL261 cells by plaque titration assay on Vero cells. Notably, viral titer gradually increased over time (Fig. 1B). By day ten post infection, the population of viable GL261 cells was markedly diminished compared to uninfected GL261 cells cultured in the same conditions (Fig. 1C). These findings underscore the susceptibility of GL261 cells to both replicative infection and killing by EGFP-oHSV-1.

An antitumoral immune response is currently recognized as a critical component of effective oncolytic virotherapy [22]. Within this framework, the induction of immunogenic cell death (ICD), which involves the release of danger-signaling molecules that activate the immune system, holds particular importance [34]. As preliminary step toward the demonstration of ICD we investigated whether oHSV-1 infection of GL261 cells could alter the levels of extracellular ATP, a key biomarker of ICD [35, 36]. Indeed, infected GL261 cells released significantly higher amounts of ATP compared to uninfected GL261 cells cultured under the same conditions (Additional File 2).

### The intratumoral administration of oHSV-1 results in rejection or strong retardation in GL261 tumor growth and acquisition of protective anti-tumor memory

**Fig. 2:**
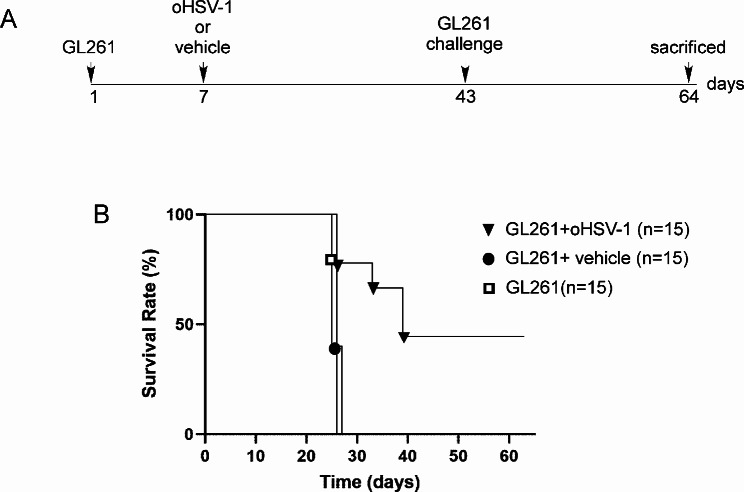
oHSV-1 treatment of GL261 tumor-bearing mice significantly improve survival. (**A**) Experimental setup and treatment schedule is depicted. (**B**) oHSV-1 intratumoral treatment (black triangles) significantly improved survival compared to animals treated with vehicle (black circles) or left untreated (white squares) with median survival of 38, 26 and 26 days respectively (*P* < 0.05). Survival was analyzed by Kaplan–Meier method and compared by log-rank Mantel-Cox test

**Fig. 3:**
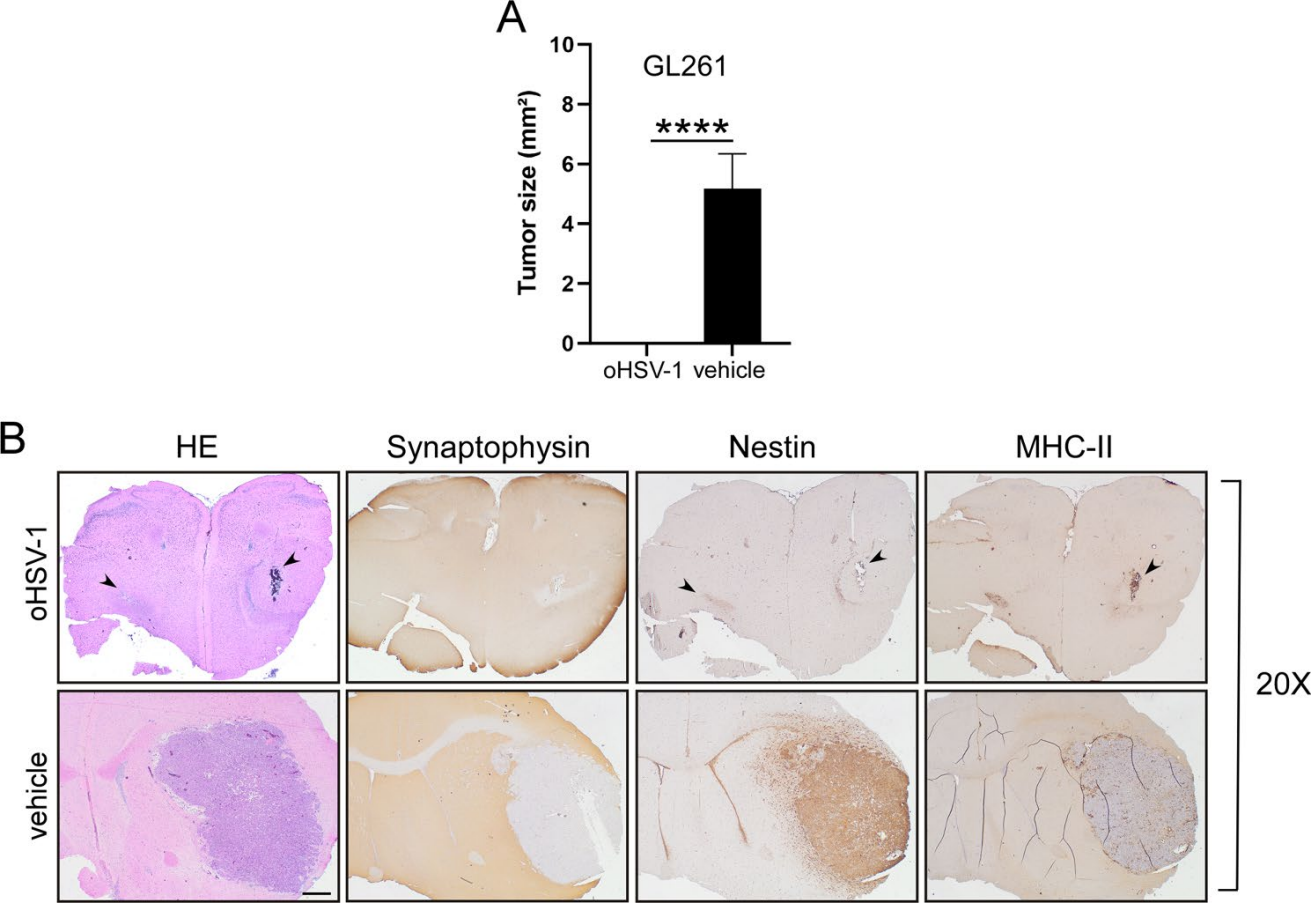
Intracranial injection of oHSV-1 in established GL261 tumor dramatically blocks tumor growth in vivo and rejects GL261 tumor challenge in the opposite brain hemisphere. C57BL/6 mice (*n* = 45) were initially injected with 3 × 10^4^ GL261 cells into the right striatum. After seven days of tumor implantation, one group of mice injected with GL261 cells was further treated with 10^6^ plaque-forming units (PFU) of oHSV-1 in the same brain hemisphere (*n* = 15). As a control, another group of mice received intracranial injections of PBS (vehicle) (*n* = 15) and the other animals were left untreated (*n* = 15). At 43 post-tumor implantation all surviving mice (*n* = 7) were challenged with parental 3 × 10^4^ GL261 tumor cells in the opposite brain hemisphere, and after additional 21 days mice were sacrificed, brains were removed, and serial sections of the brain were carried out to measure tumor size and for staining. (**A**) Average tumor size of GL261 tumor in oHSV-1 treated mice (oHSV-1) and in control group (vehicle). Data are represented as mean values, and error bars indicate the standard deviation (SD) within each group. *p*-Values were determined via unpaired t-test; ****P < 0.0001. (**B**) HE and IHC staining of serial brain sections. The panels were taken at 20x magnification. Arrowheads in HE, Nestin and MHC-II upper panels (oHSV-1) point to the sites of tumor cell injection shwing the absence of growing tumors. HE, hematoxylin and eosin; IHC, immunohistochemistry. Scale bar corresponds to 500 μM

To assess the therapeutic potential of oHSV-1 oncolytic virus in vivo we examined the effectiveness of tumor viral treatment in mice previously injected with GL261. C57BL/6 mice were initially injected with 3 × 10^4^ GL261 cells into the right striatum (*n* = 45). After seven days, one group of mice was further treated with 1 × 10^6^ PFU of oHSV-1 in the same brain hemisphere (*n* = 15). As a control, another group of mice received intracranial injections of PBS (vehicle) (*n* = 15), while a third group was left untreated (GL261) (*n* = 15) (Fig. 2A). Mice were closely monitored daily for signs of illness or distress; their weights were recorded and mice were euthanized when presenting signs of suffering. All control groups died between 25- and 27-days post-tumor injection (median survival: GL261 + vehicle: 26, GL261: 26) and at the late phase of tumor growth, showed a significant body weight loss (mean weight: GL261 + vehicle 15.2 g; GL261 14.7 g, Additional File 3). In contrast, at 43 days after tumor implantation, 45% of mice injected with oHSV-1 survived, indicating that viral treatment delayed tumor growth in *vivo* with a remarkable increase in overall survival (OS) compared to the control group (median survival: 38) (Fig. 2B). These findings strongly indicate that tumor viral treatment induces an effective tumor cell death possibly followed by antitumoral immune response responsible for rejection or strong delay in tumor growth. To further confirm the oHSV-1-mediated anti-tumor response, we investigated whether oHSV-1 tumor treatment in one brain hemisphere could prevent the growth of parental tumor cells in the opposite hemisphere. For this purpose, all surviving mice (*n* = 7) were challenged with parental GL261 tumor cells in the left striatum (Fig. 2A). After three additional weeks (day 64), the animals were sacrificed, and their brains were analyzed for the presence and size of tumors. Brain sections of representative oHSV-1-treated and non-treated mice clearly depict how oHSV-1-intratumoral injection significantly impaired the growth of GL261 parental tumor not only at the site of injection but more importantly in the opposite hemisphere with a complete tumor regression in the 100% of mice, as compared to non-treated mice, in which the average tumor size was 5.1 mm2 (average tumor size: 0 versus 5.1 mm2; unpaired Student’s t-test, *P* < 0.0001) (Fig. 3A).

**Fig. 4:**
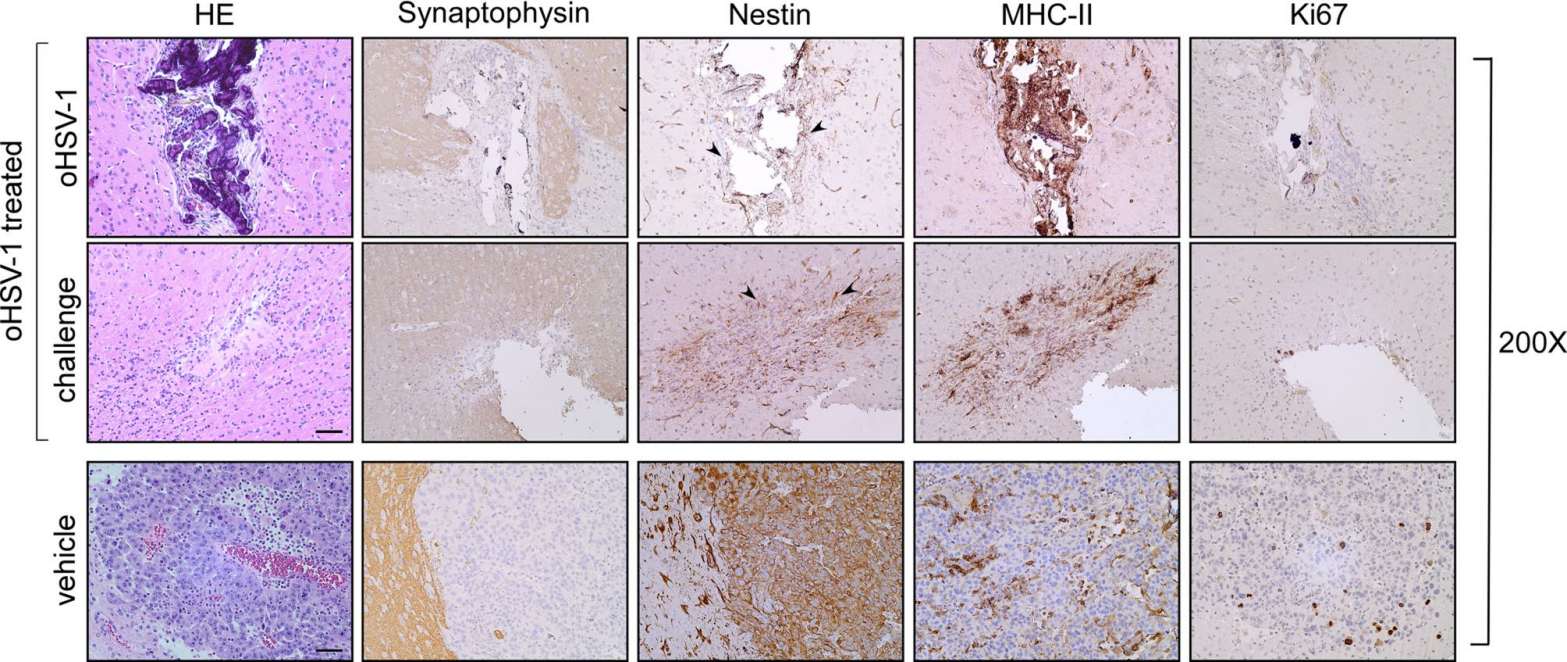
Immunohistological characterization of GL261 tumor rejection in oHSV-1 treated mice. Representative histological sections of tumor tissues harvested from pre-treated (oHSV-1 treated: oHSV-1 and challenge) and un-treated mice (vehicle) mice, at 200x magnification. HE was followed by immunohistochemical staining with synaptophysin- and nestin-specific antibodies to better identify tumoral and non-tumoral tissue, respectively. Arrowheads in the panel showing nestin staining of oHSV-1 treated mice point to astrocytosis-enriched areas in both brain hemispheres. MHC-II expression was observed on myeloid cells concentrated over the tumor bed in both brain hemispheres of oHSV-1 treated mice (oHSV-1 treated) or dispersed along the GL261 tumor (vehicle). Ki67-positive cells are abundant in tumors isolated from non-treated mice (vehicle). HE, hematoxylin and eosin. Scale bar corresponds to 50 μM

Indeed, HE-stained sections of both brain hemispheres of oHSV-1-treated mice revealed a residual tumor bed, mostly populated by fibrous cells (Fig. 3B, oHSV-1, HE, arrowheads; Fig. 4, oHSV-1 treated). These features are consistent with a complete tumor regression as compared to control untreated mice injected only with GL261 (Figs. 3B and 4, vehicle; HE). Staining for Synaptophysin, a marker of neuronal cells clearly defined the boundaries of the tumor bed (Fig. 3B, oHSV-1, Fig. 4, oHSV-1 treated; Synaptophysin), and of the tumor area in GL261-untreated mice (Figs. 3B and 4, vehicle; Synaptophysin). In non-treated mice, Nestin, a marker of neuro-epithelial stem cells not expressed in mature neurons, but expressed in activated astroglial cells and tumor cells, exhibited strong expression in GL261 parental tumors and in peritumoral astrocytes (Figs. 3B and 4; vehicle; Nestin). Interestingly, in oHSV-1-treated mice, a dense population of Nestin-positive peritumoral astrocytes was observed along the tumor bed, both in oHSV-1-treated and GL261-challenged tumors (Fig. 3B, oHSV-1, Fig. 4, oHSV-1 treated; Nestin, arrowheads).

MHC class II expression was evaluated in both treated and non-treated mice. Prominent membranous and cytoplasmic MHC class II expression was observed at the oHSV-1 injection site, accompanied by an abundant myeloid population distributed along the neoplastic bed (Fig. 4, oHSV-1, MHC-II, arrowheads). MHC-II-positive cells were also detected within peritumoral vessels. In GL261-challenged tumors, a substantial MHC-II-restricted myeloid population was distributed throughout the fibrous bed and its immediate vicinity (Fig. 4, challenge; MHC-II). Conversely, MHC-II-positive cells were observed dispersed within the GL261 tumor mass of oHSV-1 untreated mice (Figs. 3B and 4, vehicle; MHC-II).

Furthermore, the cellular proliferation rate, as indicated by Ki67 staining, demonstrated high proliferation levels in GL261 parental tumors of oHSV-1-untreated mice (Fig. 4, vehicle; Ki67). In contrast, Ki67 staining was negative in both hemispheres of oHSV-1-treated mice (Fig. 4, oHSV-1 treated; Ki67).

Taken together, these results strongly suggest that intratumoral administration of oHSV-1 in established GL261 tumors triggers an effective and durable immune response, leading to tumor regression. More significantly, it also blocks the growth of parental tumors in the opposite brain hemisphere.

### GL261-tumor rejection in oHSV-1-treated mice correlates with a strong increase in infiltrating T lymphocytes

To investigate the possible correlation between inhibition of tumor growth and immune cell infiltrate, brain sections were analyzed by IHC using T cell markers (CD3, CD4, CD8, FOXP3, TIM3 and PD-1).

**Fig. 5:**
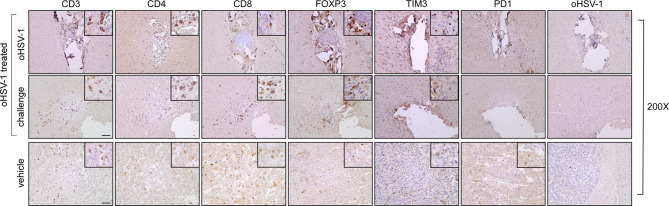
Rejected GL261 parental tumors in oHSV-1 pre-treated mice are strongly infiltrated by both CD4 + and CD8 + T cells and do not present HSV-1 viral particles. Representative immunohistology images of tumor sections. Slides of brain tissues isolated from oHSV-1-injected GL261 tumors (oHSV-1) and challenged with GL261 (challenge) are indicated in the upper and middle panels, respectively. Slides of brain tissues isolated from GL261 + vehicle (vehicle) are indicated in the lower panels. Sections were stained by IHC with anti-CD3, anti-CD4, anti-CD8, anti-FOXP3, anti-TIM3, anti-PD1, and anti-HSV-1 antibodies. Small square boxes are the areas represented in the corresponding large square boxes of each IHC image. Images were taken at 200x magnification. Large square boxes were taken at 400x magnification. IHC, immunohistochemistry. Scale bar corresponds to 50 μM

In both brain hemispheres of oHSV-1-treated mice, microscopic evaluation of immunostained sections revealed that CD4 + and CD8 + T cells were predominantly localized along the fibrotic tissue, with partial infiltration into the surrounding brain parenchyma. Both CD4 + T and CD8 + T cells were widely distributed within the tumor bed and along its margins (Fig. 5, oHSV-1 treated; CD4,CD8, insets ×400), whereas they were less abundant and dispersed throughout the GL261 tumor mass in non-treated mice (Fig. 5, vehicle; CD4, CD8, insets ×400). Interestingly, a detectable population of cells positive for FOXP3, a marker usually associated with a regulatory T cell phenotype (Treg) with inhibitory function on CD4 + T cells, was observed in both hemispheres of oHSV-1-treated mice (Fig. 5, oHSV-1 treated; FOXP3, inset ×400). Similarly, detectable TIM3 + cells were observed in both hemispheres, although to a lesser extent compared to FOXP3 + cells (Fig. 5, oHSV-1 treated; TIM3, inset ×400). On the contrary, a complete absence of PD1 + tumor-infiltrating lymphocytes was observed (Fig. 5, oHSV-1treated; PD1, insets ×400). Conversely, GL261 tumor tissue of untreated mice exhibited a lower number of infiltrating FOXP3 + cells, some TIM3 + cells and again virtual absence of PD1 + cells (Fig. 5, vehicle; FOXP3, TIM3, PD1, insets ×400).

Furthermore, to assess the correlation between tumor remission and virus replication, immunohistochemistry was used to detect viral antigen. However, no evidence of HSV-1 antigen staining was observed in all cases (Fig. 5; oHSV-1).

Taken together, these findings suggest that oHSV-1 treatment induced tumor rejection by eliciting an effective antitumoral immune response characterized by a significative recruitment of both CD4 + and CD8 + T cells, capable of protecting mice from subsequent tumor challenges.

### oHSV-1 intratumoral treatment affects the distribution and the activation of glioma-associated microglia and macrophages

**Fig. 6:**
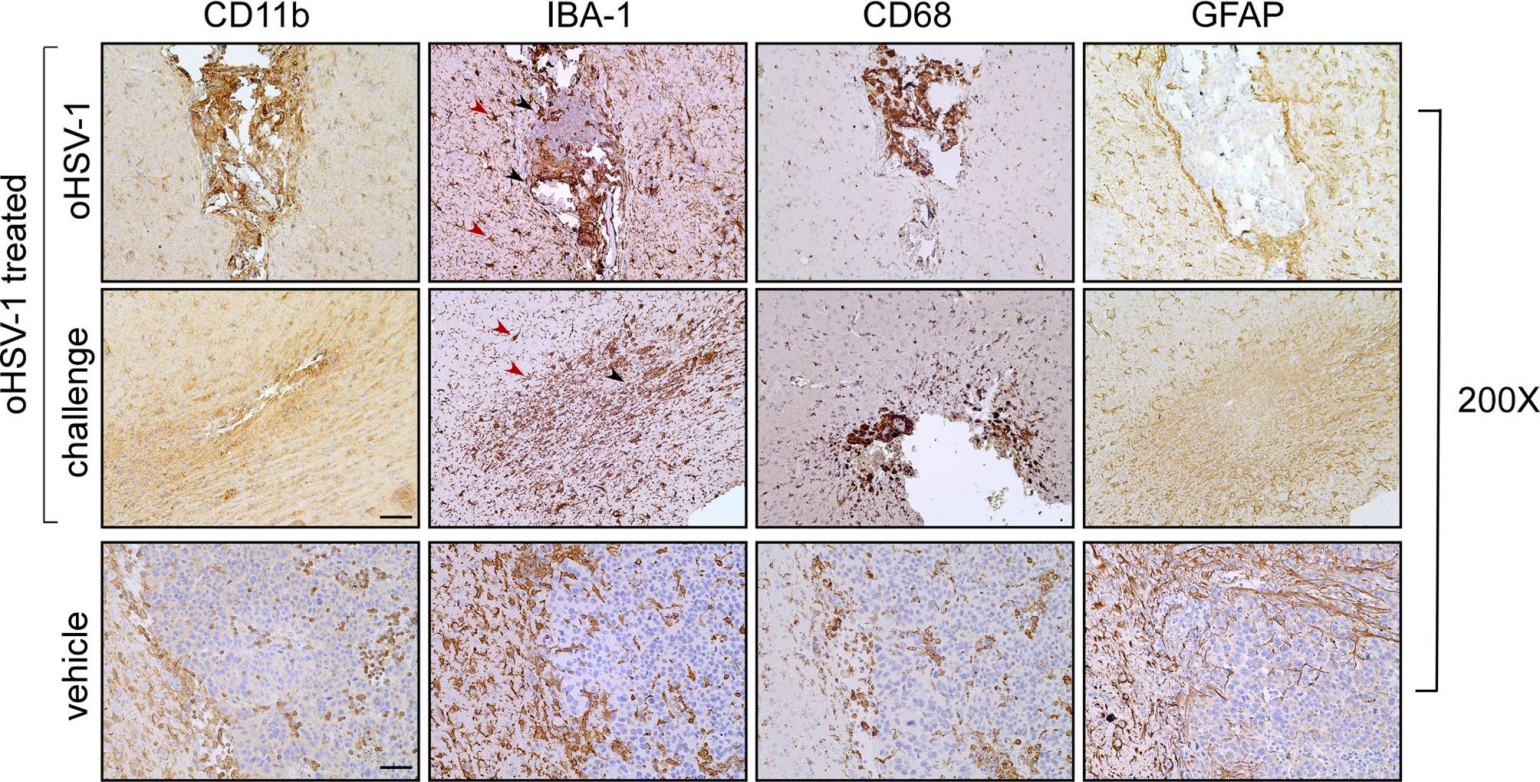
Immunohistological characterization of tumor microenvironment in GL261 C57BL/6 bearing mice treated with oHSV-1 compared to untreated mice. IHC staining of serial brain sections. Slides of brain tissues isolated from oHSV-1-injected GL261 tumors and challenged with GL261 are indicated in the upper and middle panels, respectively. Slides of brain tissues isolated from GL261-untreated mice are represented in the lower panels (vehicle). Sections were stained by IHC with anti-CD11b, anti-CD68, anti-IBA1 and anti-GFAP antibodies. Images were taken at 200x magnification. Black and red aarrowheads in the IBA-1-stained upper and middle panels point to ameboid and branched macrophages, respectively. IBA-1: Ionized calcium-binding adapter molecule 1, GFAP: glial fibrillary acidic protein. Scale bar corresponds to 50 μM

We then proceeded to evaluate the expression of specific microglia (resident and activated) and myeloid cell markers. This assessment was crucial due to the significance of glioma-associated microglia and macrophages in shaping the tumor microenvironment and recruiting tumor-infiltrating lymphocytes [37]. At the oHSV-1-treated site, we observed intense staining of CD11b + myeloid cells, most likely macrophages and dendritic cells. Similarly to the MHC-II distribution pattern, CD11b + cells predominantly infiltrated the fibrotic tissue (Fig. 6, oHSV-1; CD11b). In the challenged left hemisphere, CD11b myeloid cells were distributed along the tumor bed and diffused into the surrounding tissue (Fig. 6, challenge; CD11b). Conversely, in GL261 parental tumor, CD11b-positive cells were mainly dispersed within the tumor area and to a lesser extent around the tumor mass (Fig. 6, vehicle; CD11b). This distribution pattern resembled that observed in non-treated mice with the microglial/macrophage marker IBA-1 (Ionized calcium binding adaptor molecule 1) which is upregulated in activated microglial cells in a variety of brain diseases including GBM [38]. In this case, IBA-1 + cells were also more significantly present around the tumor mass (Fig. 6, vehicle; IBA-1). In oHSV-1-treated mice, immunostaining for IBA-1 revealed a marked increase of these cells with distinct phenotypes in both brain hemispheres. In the tumor bed, IBA-1 + cells predominantly exhibited an amoeboid shape (Fig. 6, oHSV-1 treated; IBA-1, black arrowheads), while around the tumor bed, IBA-1 + cells displayed a more branched phenotype (Fig. 6, oHSV-1 treated; IBA-1, red arrowheads). The branched and amoeboid forms are indicative of low and high activation states, respectively. Moreover, immunohistochemical staining with the CD68 pan-macrophage marker revealed a specific intense positivity in both brain hemispheres of OV-treated mice in the area of tumor cell injection, in part overlapping the area of CD11b and IBA-1 positive cells, notably, in the left hemisphere at the site of oHSV-1 injection (Fig. 6, oHSV-1; CD68), while in the opposite hemisphere, a significant population of CD68 + amoeboid cells were distributed extensively throughout the neoplastic bed (Fig. 6, challenge; CD68). Conversely, in non-treated mice, a less abundant population of CD68 + cells were dispersed over the tumor mass (Fig. 6, vehicle; CD68). Astroglial specific GFAP staining revealed distinct cellular distribution patterns between the two brain hemispheres in oHSV-1-treated mice. At the oHSV-1-treated site, astrocytic processes displayed high organization, forming a compact and dense cellular matrix lining the fibrous compartment, while GFAP + staining gradually decreased towards the healthy brain parenchyma in the surrounding area (Fig. 6, oHSV-1; GFAP). In the opposite hemisphere, the GFAP + astrocytic population exhibited an organized and dense texture around and along the tumor bed (Fig. 6, challenge; GFAP). Conversely, in non-treated mice, the GFAP + astrocytic population appeared less abundant and lacked complete organization to form a peritumoral lining (Fig. 6, vehicle; GFAP).

Taken together, these results strongly suggest that in vivo oHSV-1 treatment of GL261 reshapes the tumor microenvironment promoting the recruitment of lymphoid and myeloid cells and refining the activation profile of microglial and astroglial cells.

## Discussion

GBMs are considered immunologically “cold,” tumors [39], as they are characterized by limited T cell infiltration and a lack of expression of Human Leucocyte Antigen (HLA)-II molecules crucial for presenting tumor antigens to TH cells, the key lymphocytes that initiate the adaptive immune response. Additionally, tumor-associated macrophages (TAMs) have been observed to transition from an inflammatory phenotype to a pro-tumorigenic state, suppressing the function of tumor-infiltrating lymphocytes (TILs) rather than impeding tumor growth [40]. Consequently, most GBM immunotherapy trials have yielded only modest results [16, 17]. Thus, there is an urgent need to identify novel approaches to induce the expression of appropriate antigens and enhance the tumor immune response, rendering GBM amenable to immunotherapy. Considering that immunotherapy and conventional treatments target different aspects, combining or synergizing these approaches may lead to greater therapeutic outcomes. Replication-competent OVs are selective anticancer and immunotherapeutic agents capable of infecting, replicating within, and destroying tumor cells through multiple mechanisms without harming normal tissue. Within this frame, HSV-1-based OVs represent a promising tool. While many different oHSV-1 vectors have been proposed in the context of glioblastoma treatment, essentially three main types of oHSV-1 have been used: (1) vectors with deletions in the γ34.5 neurovirulence factor gene, which proved to be very safe in both animal and clinical studies but showed excessive attenuation in glioma stem cells [41]; (2) vectors with an additional deletion of the ribonucleotide reductase major subunit (ICP6) gene, to completely restrict viral replication to cells with active DNA replication. However, ICP6 deleted vectors showed to be so attenuated that non-tumoral cells in the tumor microenvironment prevented their spreading inside the tumor mass [42]; and (3) OVs with deletions in the γ34.5, ICP6, and US12 encoding genes. The US12 deletion enhances antigen presentation by infected cells and might increase the replication competence of the virus. An oHSV-1 with these deletions, G47∆, has been approved for the treatment of glioblastoma in Japan [21]. The viral vector we used in the present investigation, EGFP-oHSV1 (oHSV-1), has deletions of the γ34.5 and US12-encoding genes. Importantly, this is the backbone of T-VEC, which is the only OV ever approved for clinical use in the European Union and the United States, for the treatment of melanoma. The absence of the ICP6 gene deletion probably enhances replication in cancer cells compared to G47∆ and, as demonstrated here, this does not result in reduced safety after intracranial injection in mice. Our study was focussed on the efficacy of oHSV-1 in affecting the in vivo growth of a murine GBM tumor cell, GL261. Although GL261 were reported to be poorly infectable with oncolytic HSV-1 [43], we and other groups succeeded in infecting them and we observed a strong cytopathic effect in vitro [44]. The reason of the partial discordance between our data and previously reported investigations are unclear but may be due to subtle differences in the distinct susceptibility of our GL261 cell line as compared to the one of Nakashima et al. [43] and/or the greated MOI used here to infect GL261. Interestingly, cell death induced by the virus was accompanied by a strong increase of the levels of extracellular ATP, a key biomarker of immunogenic cell death. Those results motivated us to investigate the in vivo oncolytic effect of oHSV-1 and the possible triggering of an anti-tumor immune response. The first crucial observation was that mice injected orthotopically with GL261 in one brain hemisphere and subsequently treated with oHSV-1 seven days after tumor cell injection drastically improved their overall survival. In fact, 45% of animals were still alive after 64 days compared to the control group, where all mice succumbed to the disease within 26 days. To the best of our knowledge this is the first time that such results in terms of long-term survival have been obtained using a single injection of an OV in the orthotopic GL261 model, whereas comparable success has only been detected combining OVs with other immunotherapeutic treatments, such as CAR-T cells [45] or checkpoint inhibitors [46]. Although the results are already of relevance, protocols of multiple OV injections and/or different OV dosages [21] may possibly increase the protective outcome of our oHSV treatment. Future experiments will address these important questions. The success of our experimental therapeutical approach relied on the direct injection within the tumor. Peripheral intravenous inoculation of the OV instead of direct intracranial injection could be more feaseble for broader clinical application. However, this approach faces several intrinsic and biological challenges, including the neutralization of the oHSV-1 oncolytic virus by natural anti-HSV-1 antibodies present in patients, reaching and crossing the blood-brain barrier (BBB), and achieving the appropriate OV concentration within the tumor microenvironment for optimal therapeutic effect [47]. Thus, at least for now, intracranial administration appears to be a more suitable approach for treating GBM with oHSV-1. When considering the potential application of our approach in human clinical settings, several operational challenges must be addressed, foremost of which is the optimal delivery of the oncolytic virus (OV) into the tumor mass. Among the various options, newly developed technologies such as ultrasound-assisted brain delivery (UABD) technology can facilitate drug entry into the brain and enhance the therapeutic effects on brain tumors [48].

Our results suggested that oHSV-1 treatment could not only lyse tumor cells but also promote a durable, systemic antitumor response. To conclusively demonstrate the immunological basis of anti-tumor response as the cause of the extended survival, mice surviving after oHSV-1 treatment were challenged with GL261 tumor cells in the opposite hemisphere, followed for survival within a fixed time frame, after which they were sacrificed and their brains analysed by IHC. The results were dramatic, as we found that all challenged mice were tumor-free not only in the hemisphere of initial tumor injection subsequently treated with oHSV-1, but also, and more importantly, in the hemisphere receiving the GL261 challenge. Tumor rejection correlated with rapid and sustained infiltration of myeloid and particularly lymphoid cells of both CD4 + and CD8 + subtype, strongly indicating the immunological nature of rejection. Moreover, these results demonstrated two additional key features of oHSV-1 anti-GBM effect: firstly, the generation of an adaptive immune response with memory characteristics toward GBM specific tumor antigens; and second, the capacity of immune effectors to travel and act at distant sites with respect to the original site of priming, thus overcoming BBB at least within the brain. Future work will be focussed to elucidate the nature and structure of the GBM-specific stimulating antigens. An additional interesting finding of the present investigation was the presence of a substantial number of infiltrating FOXP3 + cells in the brains of oHSV-1-treated animals, both in the hemisphere originally injected with the tumor and in the opposite hemisphere receiving a GL261 tumor cell challenge. FOXP3 is a marker of Tregs, a T cell subpopulation usually associated with a negative regulatory function over CD4 + T helper cells [49]. A similar finding was previously observed by our group in mice rejecting GL261 tumor cells genetically modified to express MHC class II molecules after Class II Transactivator (CIITA) transfection [33]. The fact that in the present study, oHSV-1 treatment also results in an increased recrutitment of FOXP3 + cells suggests that these cells are not associated with an inhibitory function toward the anti-tumor response or that they were inhibited in their suppressive function by a mechanism that remains unclear, but is certainly related to the immune response triggered by the oncolytic virus. The kinetics of recruitment and accurate functional analysis of these FOXP3 + T cells certainly deserve attention in future studies.

The oHSV-1-mediated cell killing and the following protective immune response against the GBM tumor cells was accompanied by a drastic modification of the tumor microenvironment, reminiscent of similar findings in human GBM [50]. Not only did we find infiltration of bone marrow derived anti-tumor lymphocytes but also distinctive activation and redistribution of brain resident cells. Among them, resident macrophage and microglial cells were major players in the modification of the tumor microenvironment as witnessed, for example, by the intense staining for IBA-1 and CD68, markers of brain microglia and macrophages, in the tumor bed, mirror of activation and remodelling of these cellular elements. As these cells express MHC class II molecules, it is most likely that both resident macrophages and microglial cells participate to the tumor antigen presenting function necessary to stimulate tumor specific CD4 + T cells, thus triggering the adaptive immune response leading to GBM rejection. Within this frame, it is important to outline that a similar modification of tumor microenviroment was obtained by using GL261 glioblastoma cells modified to express MHC class II molecules after stable transfection of CIITA [33]. In this case GL261 themselves were acting as surrogate antigen presenting cells for their own tumor antigen for tumor specific CD4 + T cells.

An additional hallmark of the modification of the tumor microenvironment was the redistribution and activation of astroglial cell compartment. Interestingly, astroglial cells reacted differently, depending upon the specific site in which they manifested their action. In the primary tumor site corresponding to the oHSV-1 inoculation, astrocytes formed a well visible compact and dense ring of cellular matrix around the fibrous compartment that occupied the tumor area, whereas in the opposite hemisphere, site of the tumor challenge, they formed an organized and dense extended texture around and along the tumor bed, suggesting a response to distinct stimuli originated by the direct action of the virus in one site and the presence of a more extended inflammatory fibrosis in the other side. The precise cause of the astroglia distinct behaviour in the primary inflammatory versus the tumor challenge site deserves further clarification.

Taken together, the results presented here strongly support the idea that the immunosuppressive or immunoneglected glioblastoma microenvironment can indeed be modified to switch it from a “cold” to a “hot” tumor milieu by different ways, provided that sufficient inflammatory stimuli can be offered for the activation of the immune system. These certainly include the generation of danger signals at the tumor site generated by the action of a virus such as oHSV-1 [11] and, importantly, the “visibility” of sufficient amount of tumor antigens to stimulate a potent activation of a protective adaptive immune response *via* oHSV-1 tumor cell lysis. The visibility of tumor antigens can also be rescued by inducing the expression on tumor cells of MHC class II molecules after genetic modification with CIITA, as we have demonstrated in varius experimental tumor models including glioblastoma [51–53]. It is therefore tempting to speculate that an oHSV-1 virus containing CIITA may synergically increase the stimulation of the tumor specific CD4 + T cells to reach complete rejection of in vivo established glioblastoma tumors.

## Conclusions

These findings support the potential of oHSV-1 as a monotherapy for GBM, highlighting its dual mechanism of direct tumor cell killing and immune system activation. This approach may lead to new clinical strategies for GBM treatment, emphasizing the importance of immune response in enhancing therapeutic efficacy.

## Electronic supplementary material

Below is the link to the electronic supplementary material.

Supplementary Material 1

## Data Availability

The data that support the findings of this study are available from the corresponding author, upon reasonable request.

## Abbreviations

BBB: Blood Brain Barrier
GBM: Glioblastoma
IHC: Immunohistochemistry
oHSV-1: Herpes simplex virus type-1
OVs: Oncolytic viruses
TAAs: Tumor Associated Antigens
TILs: Tumor-infiltrating lymphocytes
TME: Tumor microenvironment
