# Supplementary material for: An oncolytic HSV-1 vector induces a therapeutic adaptive immune response against glioblastoma
Source: J Transl Med. 2024 Sep 27;22:862. doi: 10.1186/s12967-024-05650-5 (PMC11430576; doi:10.1186/s12967-024-05650-5)
Supplement: Supplementary file 1 — Supplementary Material 1 [file 12967_2024_5650_MOESM1_ESM.pdf]

Additional File 1

Antibody Specificity	CELL EXPRESSION	SOURCE	*(CLONE)*[A]	ANTIGEN RETRIEVAL[B]	WORKING DILUTION
**CD3**	T cells	Thermofisher (MA5-14524)	Rb (*SP7*)	E (20 min)	1/150
**CD4**	T-helper cells	Abcam (Ab183685)	Rb (*EPR19514*)	TC (20 min)	1/1000
**CD8**	Cytotoxic T-cells	Abcam (Ab209775)	Rb (*EPR20305*)	E (20 min)	1/1000
**CD11b**	Macrophages, Granulocytes and Dendritic cells	Abcam (Ab133357)	Rb (*EPR1344*)	E (10 min)	1/20000
**FOXP3**	Regulatory T-cells	Thermofisher (14-5773-82)	Rat (*FJK-165*)	TC (20 min)	1/100
**GFAP**	Astroglia cells	Genetex (GTX108711)	Rb	TC (20 min)	1/2000
**Ki67**	Proliferating cells	Abcam (Ab16667)	Rb (*SP6*)	TC (20 min)	1/100
**MHC-II**	Antigen presenting cells and CIITA-transfected tumor cells	Thermofisher (14-5321-82)	Rat (*M5/114.15.2*)	E (10 min)	1/100
**Nestin**	Neuro-epithelial stem cells, Activated Astroglia, GBM	Abcam (Ab221660)	Rb (*EPR22023*)	TC (20 min)	1/4000
**Synaptophysin**	Neurons	Abcam (Ab32127)	Rb (*YE269*)	TC (20 min)	1/6000
**IBA-1**	Pan Microglia and Macrophages	Abcam (Ab178847)	Rb (*EPR16589*)	E (10 min)	1/200
**CD68**	Macrophages and Monocytes	Abcam (Ab283654)	Rb (*EPR23917-164*)	E (10 min)	1/200
**TIM3**	Exhausted T-cells, tumor cells	Abcam (Ab241332)	Rb (*EPR22241*)	E (10 min)	1/300
**PD1**	Exhausted T-cells, tumor cells	Abcam (Ab214421)	Rb (*EPR20665*)	E (10 min)	1/300
**HSV1**	HSV-1 infected cells	Abcam (Ab9533)	Rb	TC (20 min)	1/100

[a]Rb,rabbit

[B]E, EDTA buffer pH 8.0; TC, citrate buffer pH 6.0; in parenthesis, incubation time.

Additional File 1 Antibodies used for histopathological characterization of tumor sections

Additional File 2

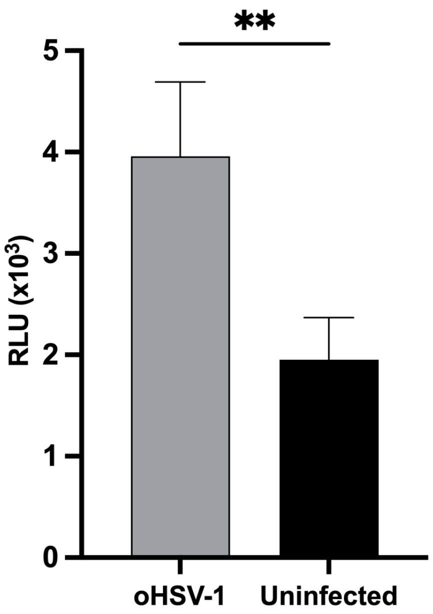

Additional File 2. oHSV-1 induces extracellular ATP release in GL261 glioblastoma cells. Cells were seeded in a white, clear-bottom 96-well plate ($5x10^3$ cells/well), infected with oHSV-1 *(MOI=5)* for 1 hour in serum-free DMEM medium, then maintained in DMEM medium supplemented with 2% FBS. Three days post-infection, extracellular ATP release was determined using the RealTime-Glo™ Extracellular ATP Assay (Promega), which yields a luminescence signal that is proportional to the amount of ATP and was measured using a Varioskan™ LUX multimode microplate reader (ThermoScientific). Uninfected GL261 cells, seeded and cultured in the same conditions for 3 days, were assayed in a similar way. The experiment was performed in 4 replicates and the difference in luminescence between uninfected and oHSV-1-infected GL261 cells was evaluated by Student's t-test. ***P=0.003*. Error bars represent standard deviation. Values on the y axis indicate real luminescence units (RLU) as measured by the instrument.

Additional File 3

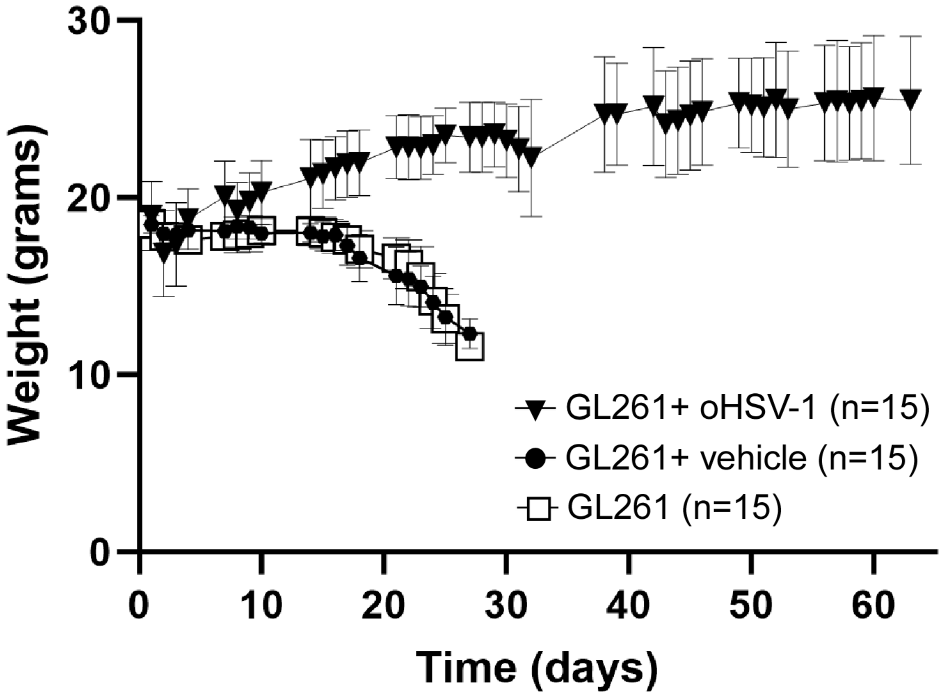

Additional File 3. Mean weight of oHSV-1- treated mice compared to control groups. Mice were weighed daily, and their weights are averaged over time. oHSV-1 intratumoral treatment (black triangles) compared to PBS (vehicle, black circles) and to parental GL261 (white squares).
